# Evaluation of knowledge and attitudes among intensive care physicians during the COVID-19 pandemic: a cross-sectional survey

**DOI:** 10.1590/1516-3180.2020.02545062020

**Published:** 2020-07-06

**Authors:** Mesut Erbas, Burhan Dost

**Affiliations:** I MD. Associate Professor, Department of Anesthesiology and Reanimation, Çanakkale University Faculty of Medicine, Çanakkale, Turkey.; II MD. Assistant Professor, Department of Anesthesiology and Reanimation, Ondokuz Mayis University Faculty of Medicine, Samsun,Turkey.

**Keywords:** COVID-19 [supplementary concept], Intensive care units, Pandemics, Airway management, Healthcare surveys, Anesthesiology, Aches Reanimation, Care unit.

## Abstract

**BACKGROUND:**

The novel coronavirus (COVID-19) emerged in Wuhan, China, in December 2019.

**OBJECTIVES:**

To evaluate the knowledge of intensive care physicians in Turkey about COVID-19 and their attitudes towards the strategies and application methods to be used for COVID-19 cases that need to be followed up in an intensive care unit, and to raise awareness about this issue.

**DESIGN AND SETTING:**

The population for this descriptive study comprised clinicians working in a variety of healthcare organizations in Turkey who provide monitoring and treatment within the intensive care process for COVID-19 patients.

**METHODS:**

Data were collected online using a survey form on the SurveyMonkey website between April 20 and April 25, 2020.

**RESULTS:**

The mean age of the 248 intensive care clinicians participating in the study was 37.2 ± 13.7 years and 49.19% were female. High rates of classical laryngoscope use were observed, especially among clinicians employed in state hospitals. Among all the participants, 54.8% stated that they were undecided about corticosteroid treatment for patients who had been intubated due to COVID-19.

**CONCLUSIONS:**

Many medications and methods are used for COVID-19 treatment. All national science committees are attempting to create standard treatment protocols. For intensive care treatment of COVID-19 patients, many factors require management, and clinicians’ experience is guiding future processes. We believe that this study will create awareness about this topic and contribute to the creation of standard treatment algorithms and the provision of better and safer healthcare services for this patient group.

## INTRODUCTION

Coronaviruses are single-chain, positive-polarity, enveloped ribonucleic acid (RNA) viruses. Their surfaces have rod-like extensions.^1^ In December 2019, it was understood that the agent causing a pneumonia epidemic in Wuhan city in China was the newly-identified severe acute respiratory syndrome coronavirus 2 (SARS-CoV-2), which was then defined as coronavirus disease 2019 (COVID-19). Patients in Wuhan with SARS-CoV-2 infection exhibited clinical symptoms over a broad spectrum from asymptomatic disease and mild tableau with mild upper respiratory tract infection to accompanying respiratory failure and severe viral pneumonia resulting in death. As a result, while some patients could be treated as outpatients, other patients required intensive care treatment.^2^^,^^3^

Currently, there is no specific treatment with proven safety and efficacy for COVID-19. Additionally, because of the urgency of the situation and the limited scientific data, treatments are commonly being chosen based on data that only show possible efficacy, for these patients around the world. Severe COVID-19 infection initially begins with flu-like complaints and is a situation that progresses to hypoxemic respiratory failure in 7-10 days. These critical patients require intensive care and this necessity is assessed by intensive care clinicians.^4^

## OBJECTIVE

In this study, we aimed to investigate the experience of clinicians who have been participating in treatments for COVID-19 patients within intensive care and their observations during the critical monitoring process for this patient group.

## METHODS

The population in this descriptive study comprised clinicians working in a variety of healthcare organizations in Turkey who provide monitoring and treatment within the intensive care process for COVID-19 patients. Permission for the study was granted by the clinical research ethics committee of Çanakkale Onsekiz Mart University (date: April 14, 2020; approval no. 2020/116). The sample size for the study was calculated using Minitab 16.0 (Minitab LLC, PA, United States). With a 95% confidence interval, 5% type 1 error and 90% power, and based on data from similar studies in terms of the parameters investigated, the power analysis determined that at least 244 people should be contacted. Data were collected online using a survey form on the SurveyMonkey website (SurveyMonkey, San Mateo, CA, United States) between April 20, 2020, and April 25, 2020. Members of the Turkish Anesthesiology and Reanimation Association were asked to participate in the study, via the Twitter, LinkedIn and WhatsApp social media platforms and through e-mail. Those who agreed to participate completed the abovementioned survey. The researchers prepared survey questions that were in line with recommendations within the COVID-19 guidelines that were published by the General Directorate of Public Health of the Turkish Ministry of Health and the World Health Organization (WHO).^5^^,^^6^^,^^7^ The survey contained 21 items: these questions related to sociodemographic characteristics, workplace features and treatment processes within intensive care that would be used in the event of a diagnosis of COVID-19.

## RESULTS

The mean age of the 248 intensive care clinicians who participated in the study was 37.2 ± 13.7 years, and 49.19% were female. Regarding the length of time for which the participants had worked in the field of anesthesia, 32.7% had worked for 1-5 years, while 24.3% had worked for 6-10 years. Evaluation of the participants’ titles showed that 56.4% were specialist doctors, while 26.6% were residents. Among the doctors who participated, 41.1% were working in universities, 30.6% in state hospitals and 16.1% in education-research hospitals. Some sociodemographic characteristics and professional information about the participants are shown in Table 1.

**Table 1.** Sociodemographic and professional characteristics of the participants (n = 248)

Variable	n (%)
Gender	
Female	126 (50.8)
Male	122 (49.1)
Age groups (years)	
21-30	40 (16.1)
31-40	96 (38.7)
41-50	84 (33.8)
51-60	26 (10,7)
61 and over	2 (0.8)
Titles	
Specialist doctor	140 (56.4)
Resident	66 (26.6)
Professor	8 (3.2)
Associate Professor	10 (4.0)
Assistant Professor	12 (9.6)
Length of time working in the field of anesthesia (years)	
1-5	80 (32.2)
6-10	72 (29)
11-15	60 (24.1)
16-20	28 (11.2)
21 or longer	8 (3.2)
Organization	
University hospital	102 (41.1)
State hospital	76 (30.6)
Education-research hospital	40 (16.1)
Private hospital	30 (12.1)

Among the clinicians working in intensive care, 50.8% stated they had received education about the COVID-19 disease from their organization. While 48.8% worked in university or education-research hospitals involved in education, 41.1% worked in state hospitals and 10.1% worked in private hospitals. Among all the participants, 87.1% worked in pandemic hospitals, and 84.6% of the clinicians considered that they had come into contact with ­COVID-19-positive patients. The proportion who thought that their workload had increased since the pandemic began was 59.6%. Among all the clinicians included in this study, 50.8% stated that COVID-19-positive patients were admitted to intensive care from the emergency service, while 44.3% stated that COVID-19-positive patients were admitted from the wards where they were being monitored.

In this study, it appeared that 45.1% of the intensive care physicians were using a videolaryngoscope, 13.7% were using an aerosol box and 41.1% were using classical laryngoscope for the intubation procedures. The proportion of intensive care clinicians stating that extubation procedures were completed at the patient’s bedside was 62.1%, while 22.5% said that extubation was done in isolated areas within intensive care and 15.3% said that extubation was done after transfer to another intensive care area. The treatment approaches and relevant opinions of the clinicians participating in the study, relating to patients who developed acute respiratory distress syndrome(ARDS) during COVID-19 treatment are shown in Table 2.

**Table 2.** Treatment approach used in relation to COVID-19 ARDS patients (%)

	Yes		No	
Do you use prone position?	77.4%		23.6%	
Do you use recruitment maneuver?	65%		35%	
Mean PEEP (cmH_2_0)	0-5	5-8	8-12	12-16
%	2.4%	32.2%	52.4%	13%

ARDS = acute respiratory distress syndrome.

When asked about the time at which favipiravir treatment (2 x 600 mg) should be used within intensive care, 63.7% of the participants stated that it should begin immediately upon the patient’s admission to intensive care, 21.7% stated that it should begin when chloroquine (2 x 200 mg) and azithromycin (4 x 250 mg) treatments were unsuccessful and 14.52% stated that it should begin when ARDS was noted in patients. The participants’ attitudes towards the interventions that are performed in intensive care units on patients infected with COVID-19 are shown in Table 3.

**Table 3.** Participants’ attitudes towards the interventions that are performed in intensive care units on patients infected with COVID-19

For suspected/confirmed COVID-19 patients, noninvasive mechanical ventilation removes the need for intubation	
Variables	%
Definitely agree	8.06
Agree	37.9
Undecided	29.8
Disagree	19.3
Definitely disagree	4.8
For suspected/confirmed COVID-19 patients, a reservoir mask is the most appropriate method for oxygen support	
Variables	%
Definitely agree	24.3
Agree	47.1
Undecided	17
Disagree	9.7
Definitely disagree	1.6
For suspected/confirmed COVID-19 patients, N95/FFP2 or N99/FFP3 masks should definitely be used during procedures that may cause aerosolization	
Variables	%
Definitely agree	94.3
Agree	4
Undecided	1.7
Disagree	0
Definitely disagree	0
For suspected/confirmed COVID-19 patients, the intubation procedure should be performed by the most experienced person	
Variables	%
Definitely agree	80.6
Agree	16.1
Undecided	1.6
Disagree	1.6
Definitely disagree	0
For suspected/confirmed COVID-19 patients, tracheostomy should be performed under planned operating room conditions	
Variables	%
Definitely agree	22.5
Agree	19.3
Undecided	26.6
Disagree	22.5
Definitely disagree	8.8
Suspected/confirmed COVID-19 patients benefit from steroid treatment	
Variables	%
Definitely agree	10.4
Agree	23.3
Undecided	54.8
Disagree	10.4
Definitely disagree	0.8
Suspected/confirmed COVID-19 patients have more incidence of super-infection during monitoring, compared with other patients	
Variables	%
Definitely agree	14.5
Agree	49.1
Undecided	22.5
Disagree	12.9
Definitely disagree	0.8
For suspected/confirmed COVID-19 cases, there is a direct correlation between mortality and advanced age for patients on mechanical ventilation	
Variables	%
Definitely agree	54.9
Agree	34.4
Undecided	5.7
Disagree	4.1
Definitely disagree	0.8

## DISCUSSION

In this study, the clinicians participating in intensive care monitoring and treatment of COVID-19-positive patients abided by the routine treatment protocols. However, different approaches were observed for some topics. When the clinical progression of COVID-19 is examined, fever, cough and dyspnea are initially observed, while in advanced cases pneumonia, respiratory failure, ARDS tableau and death are observed. Development of respiratory failure requiring intensive care in individuals infected with COVID-19 leads to a need for treatment under intensive care conditions.^5^^,^^8^ Because of the close contact between intensive care doctors and infected patients and these doctors’ potential exposure to respiratory droplets or aerosols from the patient’s respiratory tract, they are among healthcare workers with highest risk of infection.^9^

All studies have emphasized the importance of personal protective equipment (PPE) in terms of preventing transmission of infection. Especially during intubation, which produces droplets, use of N95/FFP2 or equivalent respiratory masks along with other PPE is recommended. In addition, intubation should be performed by experienced people in a single attempt if possible, using a videolaryngoscope.^10^ In our study, 45.1% of the intensive care workers used a videolaryngoscope, 13.7% used an aerosol box and the remaining 41.9% used a laryngoscope. It appeared that the clinicians with high rates of classical laryngoscope use were those working mostly in state hospitals. In a study by Dost et al.,^11^ in which the attitudes of anesthesiologists and their assistants towards patients infected with COVID-19 were investigated, theoretical and applied training that was given before they encountered the infected patients reportedly made it easier to protect both patient and healthcare worker safety, and to prevent situations of panic that would interfere with the calmness that is needed for this work.

Nearly half of the survey participants stated that high-flow oxygen and noninvasive mechanical ventilation support reduced the need for intubation. One-third of the participants stated that they were undecided about this topic. This situation may be considered to be linked to the greater chance of transmission to intensive care physicians with noninvasive ventilation. One study included a weak recommendation regarding use of noninvasive mechanical ventilation; however, it was emphasized that intubation should not be delayed.^12^

The doctors participating in our study used high positive end-expiratory pressure (PEEP) administration, suitable recruitment maneuvers and the prone position. They stated that the mean PEEP values were 8-12 cmH_2_O in ARDS cases requiring mechanical ventilation, which was in accordance with the literature. While corticosteroid treatment is not recommended for non-ARDS cases, it is recommended for cases that develop ARDS.^13^ Among all the participants, 54.8% stated that they were undecided about corticosteroid treatment for intubated patients treated in intensive care due to COVID-19.

Favipiravir (T-705; 6-fluoro-3-hydroxy-2-pyrazinecarboxamide) is an anti-viral agent that selectively and potently inhibits the RNA-dependent RNA polymerase (RdRp) of RNA viruses. It is probably effective against the RNA virus of SARS-CoV-2. Preliminary results from a study on 80 patients showed that favipiravir had a stronger antiviral effect than lopinavir/ritonavir. The favipiravir group had significantly fewer side effects than lopinavir/ritonavir. Among ­COVID-19 patients, favipiravir did not significantly improve clinical amelioration rates on the seventh day, compared with arbidol.^14^^,^^15^ The intensive care doctors participating in the present survey stated that favipiravir treatment for COVID-19 critical patients was effective if it was begun upon first admission to intensive care.

## CONCLUSION

The COVID-19 pandemic has spread rapidly around the world. Concerns about a second wave have been expressed worldwide. The incidence of COVID-19 cases around the world and in Turkey is increasing every day, and these cases may result in death. Patients developing moderate and advanced respiratory failure are admitted to intensive care units for close monitoring and treatment and require mechanical ventilation. Many drugs and methods are being used with the aim of treating the disease. National scientific committees in all countries are trying to create standard treatment protocols. Many factors require management during intensive care treatment for COVID-19 patients, and clinicians’ experience in relation to this topic will guide future procedures.

Through this study, we believe that awareness of the topic of standardization of treatment algorithms will be raised, with the aim of providing better and safer healthcare services for this patient group.
